# Mind-body Dualism: A critique from a Health Perspective**

**DOI:** 10.4103/0973-1229.77436

**Published:** 2011

**Authors:** Neeta Mehta

**Affiliations:** Associate Professor, Department of Psychology, KET’s V. G. Vaze College, Mulund East, Mumbai -400 081, India; ** *Revised and peer reviewed version of a paper read at an International Seminar on Mind, Brain, and Consciousness, Thane College Campus, Thane, India, January 13-15, 2010.*

**Keywords:** *Mind-Body Dualism*, *Cartesian Dualism*, *Cartesian Dualism and Medicine*

## Abstract

Philosophical theory about the nature of human beings has far reaching consequences on our understanding of various issues faced by them. Once taken as self-evident, it becomes the foundation on which knowledge gets built. The cause of concern is that this theoretical framework rarely gets questioned despite its inherent limitations and self-defeating consequences, leading to crisis in the concerned field. The field, which is facing crisis today, is that of medicine, and the paradigmatic stance that is responsible for the crisis is Cartesian dualism—a view that mind and body are essentially separate entities. This paper discusses Cartesian dualism in the context of the practice of medicine. Focusing more closely on how disease, health and treatment are defined through this position, the paper builds up its critique by throwing light on its accomplishments, limitations and self-defeating consequences. The paper also seeks to understand why this dualism is still alive despite its disavowal from philosophers, health practitioners and lay people.

## Introduction

Mind and body dualism represents the metaphysical stance that mind and body are two distinct substances, each with a different essential nature. Originated in the ancient period, a well-known version of dualism is credited to Rene Descartes of the 17^th^ century. According to him, human beings consisted of two quite unlike substances which could not exist in unity. Mind was unextended, an immaterial but thinking substance and body was an extended, material but unthinking substance. The body was subject to mechanical laws; however, the mind was not (Descartes, 1952). Therefore, as described by Ryle (1949), “A person… lives through two collateral histories, one comprising of what happens in and to the body, the other consisting of what happens in and to his mind… The events in the first history are events in the physical world, those in the second are events in the mental world” (p11-12).

## Mind and Body Dualism: Reformatory and Confining Force in Medicine

Mind and body dualism was the critical conceptual leap (Moon, 1995) that was desperately sought at that time in history. Before its advent, the prevalent orthodox Christian views of the mind-body relationship had greatly thwarted the development of medical science. According to these views, human beings were spiritual beings; body and soul were one. Diseases were attributed to nonmaterial forces such as personal/collective wrongdoing. It was also believed that for the soul to ascend to heaven, the human body had to be preserved intact (Walker, 1955). As a result, there was a religious prohibition on the study of human anatomy through dissection. Descartes, through mind-body dualism, demythologised body and handed over its study to medicine. Thus, the way was paved for progress in medical science through the study of physiology and anatomy. At the same time, by isolating mind, mind and body dualism denied its significance in individuals’ experience of health.

## Mind and Body Dualism: Methodological Implications

Dualism also laid the groundwork for positivism which means a logical thought based upon empirical, i.e., unbiased, impersonal and unsympathetic observation and measurement. By making objective realm the only legitimate domain of enquiry, Descartes advocated a complete and exact natural science through the analytic method. This method involved the breaking up of a problem into pieces and rearranging them in a logical order. Under the spell of the “scientific revolution” that positivism brought in, disciplines like physics, chemistry and astronomy not only flourished but also came to define exact science The success of the scientific method reinforced Descartes’ philosophy and methodology further and contributed to the dogma of scientism (Klein and Lyytinen, 1985)-the belief that scientific method was the only legitimate path to knowledge. This is an issue because disciplines under social sciences do not lend themselves to scientific method without running the risk of incomplete and at times distorted understanding of their subject matter-human beings. The field of medicine, by adhering rigidly to scientific method, mislaid its subject matter and gave up its moral responsibility toward the real health concerns of human beings.

## Mind and Body Dualism: A Basis of Biomedical Model

The dualistic stance of human nature and analytical method determined the biomedical model in medicine. Accordingly, human beings were viewed as biological organisms (materialism), to be understood by examining their constituent parts (reductionism) using the principles of anatomy, physiology, biochemistry and physics. Disease was seen as a deviation from the biological norms, caused by some identifiable physical or chemical event and intervention involved introduction of a corrective physical or chemical agent. Consequently, health came to be defined as an absence of disease and got associated with activities of doctors to the extent that to most people, medicine became synonymous with health (Hart, 1985).

## New Understanding of Human Nature and Health

Today, our understanding of human beings has changed significantly as reflected in Merleau-Ponty’s (in Gold, 1985) notion of the “lived-body” and Sprenger’s (Sprenger, 2005) summary of characteristics of living organisms. The “lived-body” notion maintains that body is not an object, but “multiphasic, experiential beings of finite freedom” (Gold, 1985, p664). It is a nucleus of one’s consciousness/intentionality. Moreover, living systems have come to be seen as systems (of which mind and body are a unit) which are integral parts of larger systems, in permanent interaction with their environment and capable of constructing their own subjective realities. These views challenge both dualistic nature of human beings and exclusive viability of positivism to pursue knowledge that is not “objective.” Simultaneously, health has also come to be viewed as something positive (Siegrist, 1941) and eventually, it received its missing dimensions when World Health Organisation (1947) defined it as a state of complete physical, mental and social well-being. More specifically, it is seen as “the capacity, relative to potential and aspirations, for living fully in the social environment” (Tarlov, 1996, p72).

In the context of this new understanding of the nature of human beings and health, the question is-how can medicine, with its narrow focus on biological factors and control of disease, help human beings achieve health which is multidimensional in nature with prevention, cure, promotion of well-being and longevity, which are proposed to be important goals of treatment? (Singh, 2010)

Emergence of diseases that have psychological, social and environmental components as part of their aetiology also challenges the hegemony of biomedicine. The consequence of this paradigmatic error is discordance between what contemporary medical professionals have got to offer and what lay people expect from them. A focus on the human body makes the field of medicine address diseases with complete disregard for illness-personal, interpersonal and cultural reactions to disease. As freedom from illness is as much needed as freedom from disease to experience health and well-being, what one finds rampant is patients’/family’s dissatisfaction with contemporary medicine. Part of dissatisfaction is also due to disempowerment of patients and dehumanisation of medical care-cold, impersonal, technical style of clinical practice shaped by notion that the body is a machine devoid of self (Kriel, 2003). Ever increasing litigation rates (Singh and Singh, 2005a), patient noncompliance, increasing resort to alternative practices, mounting consumer criticism (Kleinman, Eisenberg and Good, 1978) also reflect failure of the biomedical model to cope with lay persons’ health issues.

## Why Mind-Body Dualism is Still Alive?

As a reaction to the inadequacies of mind and body dualism, several nondualistic philosophical frameworks have been proposed. Still, mind and body dualism persists in the field of medicine. The reasons are multiple: The medical knowledge of the last 300 years is built on the biomedical model. Lot of money, energy, dedication have been invested in this field, which has paid back hugely in terms of technological success. This success has made medicine a very powerful and all encompassing health care field and has reinforced the philosophy that formed the basis of biomedical paradigm (Kriel, 2003). The pharmaceutical companies with their focus on commercial interests have great stakes in the existing medical system. They fund research in a big way but opt for status quo by selectively publishing their findings (Singh and Singh, 2005b) which does not allow new knowledge to surface. Established importance of drugs in the treatment of diseases, drug taking as a norm for any health concern and cultural tendency to expect quick remedies do not allow paradigmatic change to take place in favour of alternative and complementary medicine based on holistic view of human beings. Physicians are neither aware of the philosophical framework within which they operate, nor do they realise the power such model exerts on their thinking and behaviour. It is so because the dominant model is not necessarily made explicit, though the entire sociocultural and educational context of medical education/training reflects the prevailing conceptual model of nature of human beings, health and disease (Kriel, 2003). So strong is the influence of these philosophical frameworks that they act as blinders and human beings who are known as cognitive misers (Taylor, 1981) tend to treat them as facts and whatever does not fit into the paradigm as trivial or even nonsense. Therefore, even when unity of mind and body presents a more realistic picture of the human functioning, physicians rather stick to the familiar dualistic thinking to match that of their mentors and colleagues. Like medical practitioners, patients also perpetuate the mind and body dualism. Being a product of modern dualistic culture, they tend to feel sceptical about nonbiological explanations for their illnesses, as they appear unreal, illegitimate and unscientific in nature (Duncun, 2000).

## Concluding Remarks [see also Figure 1]

**Figure 1:**
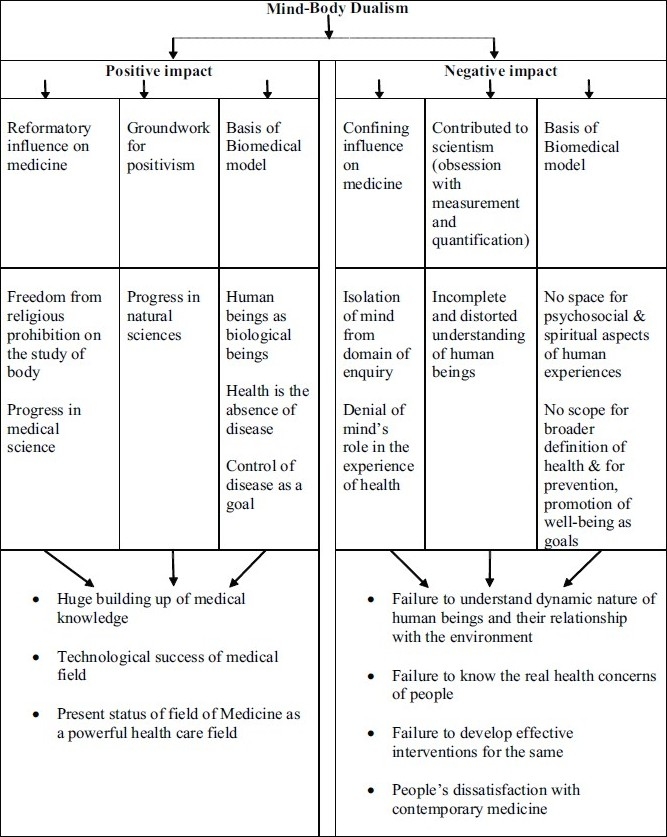
Flowchart of paper

Mind and body dualism was a convenient philosophy that used the “divide and conquer” strategy to cope with prevalent religious thinking, and subsequently fitted well to deal with the complexity of human nature. It, however, cost us dearly, as it took our focus away from the dynamic nature of human beings, their relationship with the environment and their real health concerns, and to that extent blocked the development of effective interventions. Our journey toward knowledge and understanding of nature has never been forward moving seamlessly. Mind and body dualism and its influence on medicine is a prototype of that same journey: of great strides forward and a huge leap backward.

### Take home message

Philosophical assumptions on which knowledge and practices are built need to be questioned and revised time and again for their viability; else they compromise our search for knowledge and effectiveness of practices originating therein. Mind-body dualism is an example of a metaphysical stance that was once much needed to unshackle science and medicine from dogma, but which later had far reaching restrictive influence on the field of medicine, on its complete understanding of real health issues, and on developing effective interventions to deal with the same.

## Questions That This Paper Raises

1. What kind of metaphysical stand with respect to mind and body relationship is conducive to addressing the health issues of human beings?
2. Which methods of investigation should supplement the analytical method to study all the influences on health of human beings?
3. How can one bridge the mind-body divide that is persisting despite its self-defeating consequences in the field of medicine? Where should we begin?
4. Can alternative and complementary medical practices (based on a different set of assumptions) originating in different cultures be integrated with conventional medicine to effect improved health care? If yes, how? If no, what are the obstacles?

## About the Author

*Dr. Neeta Mehta is a Clinical Psychologist by training. For the last almost two decades as Associate Professor, Department of Psychology, V.G. Vaze College (affiliated to Mumbai University) she has taught papers including Health Psychology, Human Development, Abnormal Psychology and Counselling Psychology. She has also worked as Programme Officer of National Service Scheme (N.S.S.)Unit for about five years, contributing to projects like AIDS Awareness and Gender Justice for college students. She is a member of the Ethics Committee for Scientific Research Centre conducting cosmetology research. She is the author of two books - Type A Behavior Pattern: A Risk Factor in Coronary Heart Disease and a collection of twelve skits for children. She is a contributing columnist on various psychosocial issues in a monthly publication with international circulation titled “Sailor Today.”*
